# The Associations of Job Stress and Organizational Identification with Job Satisfaction among Chinese Police Officers: The Mediating Role of Psychological Capital

**DOI:** 10.3390/ijerph121214973

**Published:** 2015-11-30

**Authors:** Lu Lu, Li Liu, Guoyuan Sui, Lie Wang

**Affiliations:** 1 China Medical University—The Queen’s University of Belfast Joint College, China Medical University, Shenyang 110122, China; zhuzhu66lulu@163.com; 2 Department of Social Medicine, School of Public Health, China Medical University, Shenyang 110122, China; liul@mail.cmu.edu.cn (L.L.); lgc0519@126.com (G.S.)

**Keywords:** job satisfaction, job stress, organizational identification, psychological capital

## Abstract

Police officers’ job satisfaction is an important issue for police force management, but insufficient research exists on the topic, especially in China. This study aimed to examine the associations of job stress and organizational identification with job satisfaction among Chinese police officers, and particularly the mediating role of psychological capital (PsyCap). A cross-sectional study was conducted in Liaoning Province of China during the period of September–October 2014. A set of self-administered questionnaires was distributed to 2514 police officers, and complete responses were obtained from 2226 participants. The associations among variables in relation to job satisfaction were validated by structural equation modeling. Job stress was negatively associated with job satisfaction, while organizational identification and PsyCap were positively associated with job satisfaction among Chinese police officers. PsyCap mediated the associations of job stress and organizational identification with job satisfaction. Interventions to improve Chinese police officers’ job satisfaction should be developed in the future, especially the enhancement of PsyCap.

## 1. Introduction

Job satisfaction is a subjective feeling about how much an individual’s needs are met by a job and can be expressed as “the extent to which people like their jobs” [1]. The basis for the assessment and investigation of job satisfaction was constructed from the Motivation-Hygiene theory [2], according to which employees’ feelings toward their jobs are affected by two factors, motivators and hygiene factors. Motivators are able to create satisfaction by satisfying the individual’s needs for personal growth and meaning, while hygiene factors can minimize the feeling of dissatisfaction. Reviews of research across a number of occupations have consistently found a link between low employee job satisfaction and high employee turnover and absenteeism, low productivity, and low organizational commitment [3]. Therefore, job satisfaction may supply as an appropriate indicator of employee effectiveness, behavior of employees, and organizational activities. Investigating job satisfaction can help emphasize factors that increase it, which can improve organizational profit and productivity accordingly [4].

Police personnel play a vital role in maintaining the disciplinary and legislative homeostasis of society. However, police work has been viewed as one of the most tiring and stressful jobs in the world [5] because of its shift work, threats of violence, excessive paperwork, confrontational contact with the public, the militaristic nature, and having to deal with a bureaucratic organizational structure [6,7]. Police management literature holds the compelling view that attaining and maintaining adequate levels of job satisfaction is necessary for the satisfactory job performance and retention of police officers, and it could also result in police organizations’ success [8,9,10,11,12]. However, empirical studies on job satisfaction and its determinants within police organizations are sparse [13], especially in China. Therefore, to investigate job satisfaction and its determinants in police officers is important for Chinese police administrators.

**Figure 1:**
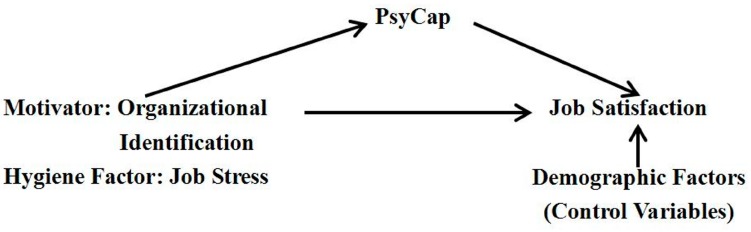
Conceptual framework of this study. Note: PsyCap: psychological capital.

The conceptual framework of this study is based on the Motivation-Hygiene theory, and is summarized in Figure 1. According to the Motivation-Hygiene theory, hygiene factors include physical working conditions, job security, benefits and so on [2]. In reviewing the literature, it is apparent that job stress is the most frequently studied contributor to job satisfaction. Job stress is described as the psychological strain or distress that arises from individual or organizational stressors in the workplace [14]. It can also result from an imbalance between the individual’s ability to cope and the demands placed on them [15] or an imbalance between employees’ efforts on the job and the subsequent rewards they receive [16]. Job stress has been recognized as one of the most important occupational hazards that can impair psychological well-being, physical health, work performance, and job satisfaction [14,17]. As job stress has been noted consistently as one of the major factors negatively affecting employees’ job satisfaction among various workplace groups [18], it may be considered as a hygiene factor affecting job satisfaction among police officers. Therefore, we formulate a first hypothesis:

H1  There is a negative association between job stress and job satisfaction among police officers.

Motivators in the Motivation-Hygiene theory include the work itself, personal achievement, recognition, and so on [2], which satisfy a person’s require for self-actualization and lead the employee to establish positive attitudes. Organizational identification is grounded in social identity theory and is a specific form of social identification where the individual defines him or herself in terms of their membership in a particular organization [19]. Individuals who identify with their organizations are likely to have positive attitudes toward them [20], contributing to better efficiencies in organizations [21]. The results of research in various occupations indicated that organizational identification was significantly associated with job satisfaction, job performance, and organizational citizenship behavior [22]. Within this framework, it can be expected that organizational identification would be considered as a motivator affecting job satisfaction among police officers. Thus, we hypothesize:

H2  There is a positive association between organizational identification and job satisfaction among police officers.

It is worth mentioning that earlier studies on job satisfaction did not pay sufficient attention to the psychological characteristics of employees. Positive psychology in recent years has focused on what it means to live the good life, and psychological capital has emerged as an important part of it. Psychological capital (PsyCap) is positive state-like psychological capacities, and focuses on people’s strength and how they grow and thrive. With the development of positive psychology, PsyCap has become an important internal resource for positive work behaviors, job attitudes (e.g., job satisfaction) and employee performance [23,24]. It has been found to be beneficial in human resource management for combating employee stress and so on [25]. Besides the research focused on the antecedents and outcomes of PsyCap, there have been other studies trying to find the underlying mechanisms through which PsyCap influences workplace outcomes at different level of analysis. Luthans *et al.* found that PsyCap fully mediated the relationship between a supportive organizational climate and job performance [26]. Some studies found the mediating role played by PsyCap in linking transformational and authentic leadership behavior to individual-level and team-level work outcomes [27,28]. Roberts *et al.* looked at PsyCap as a mediator in the association of job stress with uncivil behavior [29]. Given those prior researches, we suggest that PsyCap may influence job attitude outcomes, such as job satisfaction, in a similar way. Based on the above, we formulate the following hypotheses:

H3  PsyCap plays a mediating role in the association between job stress and job satisfaction.

H4  PsyCap plays a mediating role in the association between organizational identification and job satisfaction.

## 2. Methods

### 2.1. Study Design and Sample

A cross-sectional study was conducted during the period of September–October 2014 on a police population in Liaoning Province of China using a multi-stage cluster random sampling method. Based on the population and economic development of Liaoning Province, the 14 cities in Liaoning Province were divided into three ranks: large, medium, and small cities. Next one large city, two medium cities, and four small cities were randomly selected. Five police stations were randomly selected in each sampled city, and if the sampled city was a megalopolis, two more police stations were randomly selected. As a result, a total of 37 police stations and 2514 police officers were recruited in this study. After a description of the study was given to this population, their written informed consent was obtained. Then, a set of self-administered questionnaires was distributed to the police officers, and they completed these questionnaires anonymously. Complete responses were obtained from 2226 participants (effective response rate: 88.54%). This study received ethics approval from the Committee on Human Experimentation of China Medical University.

### 2.2. Measurement of Job Satisfaction

The Minnesota Satisfaction Questionnaire (MSQ) is a well regarded measure of job satisfaction. The 20-item short form of MSQ is a 5-point scale (from 1 = very dissatisfied to 5 = very satisfied), including two dimensions: intrinsic job satisfaction and extrinsic job satisfaction. Intrinsic job satisfaction includes 12 items that refer to activity, ability utilization, achievement, and so forth. Extrinsic job satisfaction includes six items that refer to supervision-human relations, company policies, compensation, and so forth. Intrinsic and extrinsic job satisfactions increase with higher scores. Overall job satisfaction is a total of 20 items and can be considered as a composite of all the facets of job satisfaction. The score of overall job satisfaction ranges from 20 to 100. A score of 60 indicates neutral, and a score of 80 indicates satisfied [30].

### 2.3. Measurement of Job Stress

Job stress was measured according to Siegrist’s effort-reward-imbalance (ERI) at work model in which chronic work-related stress is identified as non-reciprocity or imbalance between high efforts spent and low rewards received [31]. It is a 23-item scale that consists of three scales termed extrinsic effort (six items), reward (11 items), and overcommitment (six items), and the Chinese version of the scale has been widely used in China with good reliability and validity [32,33]. The effort and reward measurement procedure includes two steps: firstly, participants have to express their attitudes (agree or disagree) toward the work condition described by the item; then, they are asked to choose the extent to which they feel distressed (ranging from “not distressed” to “very distressed”). Overcommitment is assessed with 6-item form on a 4-point rating scale, and participants express to what extent they agree or disagree with the given statements. For the ERI scale, job stress can be expressed by effort/reward ratio (ERR) and overcommitment independently. The ERR was calculated based on the following equation: ERR = 11 × effort/6 × reward. When ERR becomes > 1, adequate reward not met with a high amount of effort is indicated. High score on overcommitment indicates the tendency to spend an inadequate amount of effort not met by externally defined reward.

### 2.4. Measurement of Organizational Identification

Organizational identification was measured by a six-item scale using 5-point rating (from 1 = strongly disagree to 5 = strongly agree), which was developed by Mael and Ashforth. It has only one dimension, and sample items include “When someone criticizes the police station, it feels like a personal insult.” and “I am very interested in what others think about the police station.” Higher scores indicate higher level of organizational identification. The scale has been widely used with good reliability and validity [19], and the Chinese version has been developed and validated in many studies [34].

### 2.5. Measurement of PsyCap

PsyCap was measured with the 24-item Psychological Capital Questionnaire (PCQ-24) which was developed by Luthans. The reliability and validity of the PCQ-24 questionnaire have been validated across multiple samples [35,36]. The PCQ-24 questionnaire consists of four dimensions: self-efficacy, hope, resilience and optimism, and each of the four dimensions is measured by six items. Each of the items is scored on a Likert scale in which 1 indicates strongly disagree and 6 indicates strongly agree. All questions ask the participants how they feel “right now”. Higher values indicate higher levels of experienced PsyCap.

### 2.6. Demographic Characteristics

Age, gender, marital status, educational level, and years of service in policing were collected in this study. Marital status was categorized as single, married/cohabitation, and divorced/separated/widowed. Educational level was categorized as high school or under, junior college and undergraduate or above.

### 2.7. Statistical Analysis

Differences in continuous variables were examined by t-tests and one-way ANOVAs. Pearson’s correlation coefficients were used to examine correlations among continuous variables. The associations among variables in relation to job satisfaction were validated by structural equation modeling (SEM). SEM is considered a more effective statistical technique than multiple regression analysis when multiple variables are acting on an outcome and interacting at the same time, and provides more insight into the direct and indirect effects [18]. Job satisfaction, job stress, organizational identification and PsyCap were all regarded as latent variables, and the control variables (age and gender) were put in the model. We set a dummy variable for gender, and “Female” was set as the reference group. To determine whether a proposed model shows a good fit to data, criteria that have been commonly used in prior research are: (1) the root mean square error of approximation (RMSEA) < 0.08; (2) the goodness-of-fit index (GFI) > 0.90; (3) the normed fit index (NFI) > 0.80; and (4) the comparative fit index (CFI) > 0.90 [23]. The chi-square-associated p value was omitted because of its sensitivity to a large sample size [37].

Baron and Kenny’s [38] technique was used for determining the mediating effect of PsyCap in the associations of job stress and organizational identification with job satisfaction. The meditation determination involves four analytical steps: (1) the independent variable (X) significantly predicts the mediating variable (M); (2) M significantly predicts the dependent variable (Y); (3) X significantly predicts Y; and (4) X significantly predicts Y with an addition of M. The effect of X on Y shrinks upon the addition of M to the model (partial mediator), or if X does not affect Y when M is added to the model, then full mediation is established. Then, the Sobel test was used to detect whether the mediating effect reached a significant level. In this study, X includes job stress, organizational identification, and control variables; M is PsyCap, and Y is job satisfaction.

All the continuous variables were centralized in order to avoid multicollinearity before the model was validated. Moreover, tolerance and variance inflation factor were used to check for multicollinearity. All analyses were performed using SPSS 17.0 (SPSS China Corp, Shanghai, China) and Amos 6.0 (SPSS Inc, Chicago, IL, USA). Statistical significance was defined as *p* < 0.05 (two-tailed).

## 3. Results

### 3.1. Demographic Characteristics

Demographic characteristics of subjects and distributions of job satisfaction in categorical items are shown in Table 1.

**Table 1:** Demographic characteristics of subjects and distributions of job satisfaction.

Variables	N (%)	Intrinsic Job Satisfaction	Extrinsic Job Satisfaction	Overall Job Satisfaction
		Mean (SD)	Mean (SD)	Mean (SD)
Age (years)		*p* < 0.01	*p* < 0.01	*p* < 0.01
≤34	768 (34.50)	39.91 (9.58)	20.18 (5.01)	66.96 (15.82)
35–44	937 (42.09)	38.69 (7.75)	19.48 (4.26)	64.88 (13.07)
≥45	521 (23.41)	41.11 (8.54)	19.95 (4.63)	67.85 (14.29)
Gender		*p* < 0.01	*p* > 0.05	*p* < 0.05
Male	1882 (84.55)	39.43 (8.63)	19.76 (4.59)	65.98 (14.32)
Female	344 (15.45)	41.01 (8.68)	20.22 (4.80)	68.00 (14.73)
Marital status		*p* < 0.01	*p* < 0.01	*p* < 0.01
Single	292 (13.12)	40.43 (9.37)	20.75 (4.92)	68.08 (15.39)
Married/Cohabitation	1848 (83.02)	39.38 (8.50)	19.62 (4.56)	65.75 (14.19)
Divorced/ Separated/Widowed	86 (3.86)	43.55 (8.33)	21.35 (4.51)	72.01 (14.40)
Educational level		*p* > 0.05	*p* < 0.01	*p* > 0.05
High school or under	105 (4.72)	39.79 (4.53)	21.37 (2.46)	68.78 (7.44)
Junior College	616 (27.67)	40.06 (8.80)	19.94 (4.66)	66.80 (14.60)
Undergraduate or above	1505 (67.61)	39.51 (8.81)	19.68 (4.71)	65.91 (14.67)
Years of service in policing		*p* < 0.05	*p* < 0.01	*p* < 0.05
≤10	737 (33.11)	39.77 (9.85)	20.16 (5.16)	66.80 (16.24)
11–20	867 (38.95)	39.16 (8.14)	19.42 (4.37)	65.22 (13.66)
≥21	622 (27.94)	40.27 (8.65)	20.01 (4.26)	67.20 (12.95)

Note: SD: standard deviation.

Mean intrinsic, extrinsic and overall job satisfactions differed across age groups, marital status groups and years of service in policing groups. Mean intrinsic and overall job satisfactions differed between gender groups. Mean extrinsic job satisfaction differed across educational level groups.

### 3.2. Descriptions of Job Stress, Organizational Identification, PsyCap and Job Satisfaction

The means, standard deviations, and reliabilities (Cronbach’s alpha) for job stress, organizational identification, PsyCap and job satisfaction in the study are shown in Table 2. In the study subjects, 37% reported a high amount of effort not met with adequate reward (ERR > 1). The mean score of overall job satisfaction in police officers was just medium (66.29 ± 14.40) and only 21.2% of police officers expressed satisfaction with their police job.

**Table 2:** Descriptions of job stress, organizational identification, PsyCap and job satisfaction.

Measure	Items	Mean	SD	Cronbach’s Alpha
Job stress	23	-	-	-
Extrinsic effort	6	18.12	6.54	0.95
Reward	11	37.98	10.04	0.93
Overcommitment	6	15.88	3.20	0.81
Organizational identification	6	3.49	0.94	0.94
PsyCap	24	3.98	0.85	0.96
Self-efficacy	6	4.00	1.33	0.97
Hope	6	4.13	0.99	0.89
Resilience	6	3.98	0.86	0.84
Optimism	6	3.83	0.70	0.90
Job satisfaction	20	66.29	14.40	0.97
Intrinsic job satisfaction	12	39.67	8.65	0.95
Extrinsic job satisfaction	6	19.83	4.63	0.91

Notes: PsyCap: psychological capital; SD: standard deviation.

### 3.3. Correlations among Job Stress, Organizational Identification, PsyCap and Job Satisfaction

Results of Pearson correlation are shown in Table 3. Job stress was negatively correlated with intrinsic, extrinsic and overall job satisfactions. Organizational identification and PsyCap were positively correlated with intrinsic, extrinsic and overall job satisfactions.

**Table 3:** Correlations among job stress, organizational identification, PsyCap and job satisfaction.

Variable	1	2	3	4	5	6	7
1. Age							
2. ERR	0.144 **						
3. Overcommitment	0.058 **	0.510 **					
4. Organizational identification	−0.007	−0.060 **	0.169 **				
5. PsyCap	−0.068 **	−0.096 **	0.103 **	0.555 **			
6. Intrinsic job satisfaction	0.043 *	−0.194 **	−0.089 **	0.441 **	0.545 **		
7. Extrinsic job satisfaction	−0.006	−0.197 **	−0.138 **	0.374 **	0.449 **	0.900 **	
8. Overall job satisfaction	0.024	−0.201 **	−0.111 **	0.426 **	0.514 **	0.983 **	0.959 **

Notes: ERR: effort/reward ratio; PsyCap: psychological capital; * *p* < 0.05; ** *p* < 0.01.

### 3.4. Results of SEM and the Mediating Role of PsyCap

**Figure 2:**
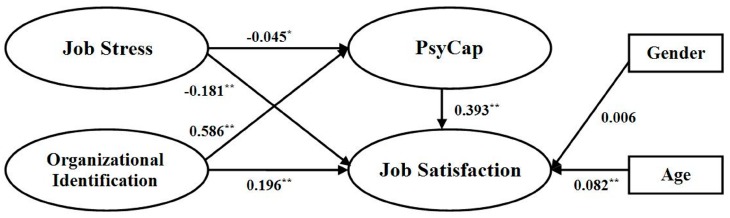
The final model with the mediating effect of PsyCap. Note: PsyCap: psychological capital; * *p* < 0.05; ** *p* < 0.01.

The four models according to the steps determining the mediating effect of PsyCap were validated for goodness of fit, and the goodness-of-fit indices in the four models all fell within the acceptable range (Table 4). The final model with the mediating effect of PsyCap is shown in Figure 2. As shown in the figure, job stress showed a negative and significant association with job satisfaction through PsyCap (β = −0.181, *p* < 0.01); while organizational identification showed a positive and significant association with job satisfaction through PsyCap (β = 0.196, *p* < 0.01). Of the two control variables, age was significantly related to job satisfaction (β = 0.082, *p* < 0.01), which indicated the younger police officers reported lower level of job satisfaction to their police jobs. The standardized squared correlations (R^2^) showed that job stress, organizational identification, and PsyCap accounted for 33% of variance in job satisfaction.

**Table 4:** The goodness-of-fit indices in the four models determining the mediating effect of PsyCap.

Model	RMSEA	GFI	NFI	CFI
X-M (Step 1)	0.08	0.94	0.95	0.95
M-Y (Step 2)	0.08	0.98	0.98	0.98
X-Y without M (Step 3)	0.07	0.96	0.96	0.97
X-Y with M (Step 4)	0.08	0.93	0.95	0.95

Notes: PsyCap: psychological capital; RMSEA: the root mean square error of approximation; GFI: the goodness-of-fit index; NFI: the normed fit index; CFI: the comparative fit index.

Regarding the mediating role of PsyCap, according to the criteria proposed by Baron and Kenny, job stress and organizational identification all consistently met the four steps required for the mediating effect. As shown in Table 5, PsyCap had a partially mediating effect in the association of job stress with job satisfaction as the standardized coefficient for job stress shrank when PsyCap was added (from β = −0.221 to β = −0.181; Sobel test, z = −2.27, *p* < 0.05). PsyCap had a partially mediating effect in the association of organizational identification with job satisfaction as the standardized coefficient for organizational identification shrank when PsyCap was added (from β = 0.448 to β = 0.196; Sobel test, z = 14.73, *p* < 0.01).

**Table 5:** Results of the proposed paths in the four models examining the mediating effect of PsyCap.

The Proposed Path	Standardized Coefficient (β)
X-M (Step 1)	
Job stress→PsyCap	−0.027 *
Organizational identification→PsyCap	0.585 **
Age→PsyCap	−0.066 **
Gender→PsyCap	−0.056 **
M-Y (Step 2)	
PsyCap→Job satisfaction	0.472 **
X-Y without M (Step 3)	
Job stress→Job satisfaction	−0.221 **
Organizational identification→Job satisfaction	0.448 **
Age→Job satisfaction	0.082 **
Gender→Job satisfaction	−0.022
X-Y with M (Step 4)	
Job stress→Job satisfaction	−0.181 *
Organizational identification→Job satisfaction	0.196 **
Age→Job satisfaction	0.082 **
Gender→Job satisfaction	0.006

Notes: PsyCap: psychological capital; Gender means “Male” *vs.* “Female”. * *p* < 0.05; ** *p* < 0.01.

## 4. Discussion

Job satisfaction has a deep knowledge base and theoretical foundation, and may be the most widely studied variables in organizational culture, behavior and other occupational phenomena [39]. However, compared with other occupations, studies on police officers’ job satisfaction are relatively limited. Moreover, it needs to be emphasized that most of the past researches on police officers’ job satisfaction were conducted in English-speaking countries in the Western world. Relatively few studies were conducted in the Eastern world. In the present study, subjects were selected from police stations in cities of every size in Liaoning Province, China. A large sample size and a higher effective response rate may be able to display a good representation of the study population and enhance the generalization of our study conclusions.

According to the results of this study, only 21.2% of police officers expressed satisfaction with their police job, and the mean score of overall job satisfaction in police officers was just medium, similar to other studies [40]. Besides, age was found to be positively correlated with job satisfaction among police officers, consistent with some studies [41,42,43]. Other studies, however, reached contradictory conclusions [44,45,46], indicating the inconclusive nature of relationships between age and job satisfaction among police officers. Moreover, in this study, 37% of police officers reported experiencing job stress, which is a higher proportion than reported in Italian police officers [47,48]. Compared with other countries, police work in China is arguably more onerous because of excess workload, constant risk of being injured due to strict gun-control regulation, inconsistency in implementing orders according to law, being the representative of the government in the eye of the public, and having to maintain social stability in a multinational region [49]. The probable reason for the higher proportion maybe related to the nature of police work, but also implied that stress-management programs are lacking in Chinese police force.

In the analyses carried out, job stress and organizational identification demonstrated their importance in relation to police officers’ job satisfaction. Our findings supported H1 and H2 that job stress had a significantly negative impact, while organizational identification had a significantly positive impact on job satisfaction among police officers, consisted with findings in other studies [50,51]. Nevertheless, organizational identification was found to have more impact on job satisfaction than job stress in police officers, but its importance in affecting job satisfaction has not been widely detected in the previous literature review. Police officers who have strong organizational identification may perceive neutral or even negative conditions as less detrimental because they are more likely to see the necessity of these conditions in order to achieve the organization’s overall goals. Furthermore, they may perceive their job as proof of their organizational membership, and therefore as validating those parts of their self that stem from this membership [50]. It is important to see the benefits of improvement in organizational identification, as this consequence will result in the improvement of job satisfaction.

Besides providing support for previous findings, our study makes the important attempt to integrate PsyCap in explaining the association between job stress and job satisfaction, as well as the association between organizational identification and job satisfaction, which is the major contribution. Our results supported H3 and H4 that: (1) job stress might affect risk for job satisfaction in police officers via a mediating mechanism of PsyCap. In other words, police officers who faced more job stress would be more likely to experience lower levels of PsyCap, which in turn would lead to lower job satisfaction. (2) Organizational identification might be a protective factor for job satisfaction in police officers via a mediating mechanism of PsyCap. That is to say, police officers with more organizational identification would be more likely to experience higher levels of PsyCap, which in turn would lead to higher job satisfaction. As an identified positive resource to fight against stress and enhance positive work attitudes, PsyCap can enhance employees’ positive appraisals of their circumstances and increase their perceived probability of success based on their motivation, effort and perseverance [52]. Accordingly, low PsyCap would cause negative expectancies and appraisals, reduced intrinsic motivation and result in more emphasis on extrinsic rewards, e.g., pay, working conditions and job security [53]. As Luthans *et al.* have suggested, employees with higher levels of overall PsyCap had greater job satisfaction and performed better at work than employees who only exhibit one component of PsyCap (self-efficacy, hope, resilience, and optimism) [52], it is crucial to enhance the four components of PsyCap in police officers for the purpose of improving job satisfaction.

Our findings have theoretical implications as well as practical implications for human resources management and performance management. In theory, the present study revealed that PsyCap might be a positive resource for combating job stress and facilitating organizational identification to improve job satisfaction. Practically, our findings should urge police administrators in China to be aware of the low levels of job satisfaction in police officers. Efforts should be made to develop strategies to decrease police officers’ job stress, and improve organizational identification and PsyCap. However, due to the special nature of the police work, it is difficult to change the job stress in police officers immediately. Moreover, investigations about how to manage organizational identification in the literature are meager. It is a more positive and feasible strategy to develop programs increasing PsyCap of police officers, thus to improve job satisfaction in a long run. Interventions designed to enhance the four components of PsyCap (self-efficacy, hope, resilience, and optimism) have been introduced in other studies and confirmed to be effective [54,55,56]. Therefore, strategies of enhancing police officers’ PsyCap should be developed in China as soon as possible in the future. Finally, our study provides a new perspective for researchers to integrate the positive psychological resources of self-efficacy, hope, optimism, and resilience to enhance job satisfaction.

Several limitations of the present study have to be mentioned. First, this study is the cross-sectional design. Job stress, organizational identification, PsyCap and job satisfaction were measured simultaneously, so causal conclusions cannot be drawn. All findings obtained in the current study should be confirmed by a longitudinal study. Second, all data were self-reported, which may introduce bias. Third, this study only focused on several variables associated with job satisfaction. The explanatory power was just medium (33% for job satisfaction), which implied that there might be other variables that impact police officers’ job satisfaction. More factors should be investigated in further studies.

## 5. Conclusions

Our findings supported the Motivation-Hygiene theory as an explanatory framework by which job stress and organizational identification impact job satisfaction. Furthermore, PsyCap was evidenced to play a mediating role in the association between job stress and job satisfaction, as well as the association between organizational identification and job satisfaction. Interventions to improve Chinese police officers’ job satisfaction should be developed in the future, especially aimed at the enhancement of PsyCap.
